# Circulating CD34+ cells and active arterial wall thickening among elderly men: A prospective study

**DOI:** 10.1038/s41598-020-61475-4

**Published:** 2020-03-13

**Authors:** Yuji Shimizu, Shin-Ya Kawashiri, Kairi Kiyoura, Jun Koyamatsu, Shoichi Fukui, Mami Tamai, Kenichi Nobusue, Hirotomo Yamanashi, Yasuhiro Nagata, Takahiro Maeda

**Affiliations:** 1 Department of Community Medicine, Nagasaki University Graduate School of Biomedical Sciences, Nagasaki, Japan; 2 Department of Cardiovascular Disease Prevention, Osaka Center for Cancer and Cardiovascular Disease Prevention, Osaka, Japan; 3 Department of Island and Community Medicine, Nagasaki University Graduate School of Biomedical Sciences, Nagasaki, Japan; 4 Department of Immunology and Rheumatology, Nagasaki University Graduate School of Biomedical Sciences, Nagasaki, Japan; 5 Department of General Medicine, Nagasaki University Graduate School of Biomedical Sciences, Nagasaki, Japan; 6 Center for Comprehensive Community Care Education, Nagasaki University Graduate School of Biomedical Sciences, Nagasaki, Japan

**Keywords:** Predictive markers, Risk factors

## Abstract

Age-related physical changes, such as low-grade inflammation and increased oxidative stress, induce endothelial repair and cause active arterial wall thickening by stimulating the production of CD34+ cells (the principal mediators of atherosclerosis). Despite this, aggressive endothelial repair (progressing atherosclerosis) might cause a wasting reduction in CD34+ cells, which could result in a lower capacity of endothelial repair and hypertension. As yet, no prospective study has clarified the association of circulating CD34+ cells with active arterial wall thickening. We conducted a prospective study of 363 men aged 60–69 years who participated in a general health check-up at least twice from 2014–2017. The circulating CD34+ cell count was significantly positively associated with active arterial wall thickening among subjects without hypertension (n = 236), but not among subjects with hypertension (n = 127). The fully adjusted odds ratios (ORs) of active arterial wall thickening for the logarithmic circulating CD34+ cell count were 1.83 (1.19, 2.84) and 0.69 (0.36, 1.32) for subjects without and with hypertension, respectively. Circulating CD34+ cells are positively associated with active arterial wall thickening in subjects without hypertension. This study demonstrates a means to clarify the mechanisms of endothelial repair in elderly subjects.

## Introduction

Recently, bone marrow-derived hematopoietic stem cells (immature cells, such as CD34+ cells) have been revealed to play a major role in vascular homeostasis^1–3^. It is likely that the number of circulating CD34+ cells could indicate the capacity of endothelial maintenance^4–6^. However, it is known that hematopoietic bone marrow activity declines with age^7–10^, which might induce a lower capacity of endothelial maintenance in elderly subjects. Furthermore, aging is a well-known cause of endothelial injury^11,12^ which subsequently induces endothelial repair. In addition, the aggressive endothelial repair that causes atherosclerosis might induce a wasting reduction of circulating CD34+ cells^6,13^ which result in a shortage of the mediators of endothelial repair. Moreover, inadequate endothelial repair might cause hypertension in elderly individuals^14^. These previous studies indicate that there are close connections among the age-related decline of bone marrow activity, age-related endothelial injury, and the wasting reduction of circulating CD34+ cells.

Platelets are also known to play an important role in vascular inflammation and vessel wall remodeling^15,16^. Indeed, platelets promote the mobilization of bone marrow-derived CD34+ cells into the peripheral blood^17–21^, and CD34+ cells also induce platelet elevation^22,23^. Furthermore, platelets have been reported to play an important role in the initial development of atherosclerotic lesions^24^, and not only induce the differentiation of human CD34+ cells into endothelial cells, but also into foam cells, which contribute to the development of atherosclerosis^25^. Therefore, platelets also play an important role in the abovementioned connection between the age-related decline in bone marrow activity, age-related endothelial injury, and the wasting reduction of circulating CD34+ cells.

Since active atherosclerosis (arterial wall thickening) caused by aggressive repair of endothelium might have a beneficial influence on the microcirculation and help prevent hypertension in elderly subjects^14,26,27^, clarifying the underlying mechanism of endothelium repair is an effective way to determine an efficient strategy for preventing age-related disease.

In a previous cross-sectional study, the beneficial association of elevated circulating CD34+ cells on endothelial maintenance were revealed to be masked by hypertension, which might be associated with a reduction in CD34+ cell levels^5,6^. Therefore, we hypothesized that the level of circulating CD34+ cells is positively associated with active arterial wall thickening in the elderly, but only in those without hypertension.

In order to clarify this hypothesis, we conducted a 2-year (2.20 ± 0.53) follow-up study of 363 Japanese men aged 60–69 years who participated in a general health check-up at least twice from 2014–2017.

## Materials and Methods

### Study population

In 2015, the total number of male residents age 60–69 years (estimated by the National Institute of Population and Social Security Research in March 2013) was 3,264 for Goto city and 1,010 for Saza town^28^.

The study population comprised 498 male residents aged 60–69 years from Goto city and Saza town, the Western rural communities of Japan, who underwent an annual medical check-up from 2014–2015 for baseline. Subjects without data for CD34+ cells (n = 2) and serum (n = 2) were excluded from the study population. In order to avoid the influence of chronic inflammatory disease, subjects with a high white blood cell count (WBC ≥ 10,000 cells/μL) (n = 5) were excluded. We also excluded 126 subjects who did not take an annual health check-up during the follow-up period (2016 to 2017). The remaining participants, 363 men with a mean age of 65.4 years (standard deviation [SD], 2.6; range, 60–69) were enrolled in the study. The follow-up period of the present study was 2.20 ± 0.53 years.

All procedures performed in studies involving human participants were in accordance with the ethical standards of the institutional research committee and with the 1964 declaration of Helsinki, and its later amendments, for comparable ethical standards. Ethical approval was obtained by The Ethics Committee for Human Use of Nagasaki University, and the study was approved by the Ethics Committee of Nagasaki University Graduate School of Biomedical Sciences (project registration number: 14051404). Prior to participate in the study, the participants provided informed consent by using written consent forms available in Japanese.

### Data collection and laboratory measurements

Medical history data were acquired by specially trained interviewers. An automatic body composition analyzer (BF-220; Tanita, Tokyo, Japan) calculated the body mass index (BMI, kg/m^2^) by measuring the height and weight. Current drinker was defined as individuals with an ethanol intake of ≥23 g/week. After at least 5 min or rest, blood pressure (systolic and diastolic) was measured in the sitting position using a blood pressure measuring device (HEM-907; Omron, Kyoto, Japan). A heparin sodium tube, an EDTA-2K tube, a siliconized tube, and a sodium fluoride tube were used to collect fasting blood samples for laboratory measurements. The heparin sodium tube was used to count the number of CD34+ cells, the EDTA-2K tube was used to measure the WBC and platelet count, the siliconized tube was used to measure high-density lipoprotein cholesterol (HDLc), triglyceride (TG) levels and the serum creatinine, and sodium fluoride tube was used to measure hemoglobin A1c (HbA_1c_). To measure CD34+ cell counts, an automated software on the BD (Beckton Dickinson Biosciences) FACSCant^TM^ II system was used in accordance with the International Society of Hematotherapy and Graft Engineering (ISHAGE) guidelines^29^. Using the standard laboratory procedure, WBC and platelet counts as well as HDLc, TG, serum creatinine, and HbA_1c_ levels were measured at SRL, Inc. (Tokyo, Japan).

Using the method proposed by a working group of the Japanese Chronic Kidney Disease Initiative^30^, the glomerular filtration rate (GFR) was calculated: GFR (ml/min/1.73 m^2^) = 194 × (serum creatinine (enzyme method))^−1.094^ × (age)^−0.287^.

An experienced vascular examiner measured both left- and right-sided carotid intima-media thickness (CIMT) of the common carotid arteries using LOGIQ Book XP with a 10-MHz transducer (GE Healthcare, Milwaukee, WI, USA). Active endothelial repair that is associated with atherosclerosis, evaluated using the maximum values of CIMT, could have a beneficial association with hypertension among subjects with high CD34+ cell levels^14^. The present study aimed to clarify the hypertension status-specific association between active arterial wall thickening (active endothelial repair) and CD34+ cell levels. Maximum values for the left and right common CIMT were then calculated using automated digital edge-detection software (Intimascope; MediaCross, Tokyo, Japan), using a previously described protocol^31^. The recently developed Intimascope software was used to increase the accuracy and reproducibility of CIMT measurement values. Semi-automatically, this software recognizes the edges of the internal and external membranes of the artery and automatically determines the distance at a sub-pixel level (estimated to be 0.01 mm)^32^. Using a part of the study population (n = 25), the reproducibility of the CIMT measurement was measured using Intimascope; this reproducibility had a satisfactory high value. We evaluated intra-observer variations (assessed by two examiners) for CIMT measurements by calculating the simple correlation coefficients between repeated CIMT measurement values and found r = 0.97 (p < 0.001) and r = 0.98 (p < 0.001). The inter-observer variation between those two examiners was r = 0.80 (p < 0.001).

### Statistical analysis

To evaluate the active arterial wall thickening, we calculated the delta values of the CIMT per year. The CIMT value is usually evaluated using visual operation under B-scope ultrasonography. Due to technical difficulties in ensuring accuracy and reproducibility, the axial resolution in this system is low (≥0.1 mm).

An innovative measurement software, Intimascope (Media Cross Co. Ltd, Tokyo, Japan), was developed to improve the axial resolution of the CIMT measurement accuracy and reproducibility using B-scope carotid artery ultrasonography. The axial resolution (an estimated scale of 0.01 mm) was found to be 10-fold higher than the usual CIMT measurement values when using this software^32^. Given that the present study used Intimascope, we defined active arterial wall thickening as increased values of CIMT ≥ 0.01 mm/year. Furthermore, baseline subclinical atherosclerosis was diagnosed as a CIMT of ≥1.1 mm because a normal CIMT value was reported as <1.1 mm in a previous study^33^.

Hypertension masks the beneficial effect of CD34+ cells^5,34^ and low levels of CD34+ cells acts as an indicator of the activity of the vicious cycle that exists between hypertension and endothelial dysfunction as evaluated using CIMT^6^. Since the present study aimed to clarify the influence of CD34+ cells on active arterial wall thickening, hypertension status-specific analyses were performed. We defined hypertension according to previous studies^5,6,34^, namely, systolic blood pressure ≥140 mmHg and/or diastolic blood pressure ≥90 mmHg. The characteristics of the study population in terms of hypertension status were expressed as mean ± standard deviation for normally distributed contentious variables. The number of CD34+ cells and triglycerides were expressed as the median (first and third quartiles) because these values show skewed distributions. Moreover, medication usage and status (current drinker and current smoker) were expressed as percentages.

Logistic regression models were used to calculate odds ratios (ORs) and 95% confidence intervals (CIs) to determine the association of hypertension with baseline subclinical atherosclerosis and active arterial wall thickening. We also calculated the ORs and 95% CIs for active arterial wall thickening in relation to baseline subclinical atherosclerosis by using a logistic regression model. Furthermore, hypertension status specific ORs and 95% CIs for active arterial wall thickening, in relation to circulating CD34+ cells, were also calculated.

Three different models were used to calculate the ORs and 95% CI. In the first model, the only adjustment was for age (Model 1). For Model 2, further adjustments were made for smoking status (never smoker, former smoker, or current smoker). Finally, Model 3 was further adjusted for other potential confounding factors, namely systolic blood pressure (mmHg) (systolic blood pressure was not adjusted for the analysis to determine the association of hypertension on baseline subclinical atherosclerosis and active arterial wall thickening), BMI (kg/m^2^), alcohol consumption (never drinker, former drinker, or current drinker [23–45 g/week, 46–68 g/week, ≥ 69 g/week]), HDLc (mg/dL), triglycerides (mg/dL), HbA1c (%), and GFR (mL/min/1.73 m^2^). Those confounding factors were the same as a previous study that examined CD34+ cells, hypertension, and atherosclerosis^14,35^. We also performed a simple correlation analysis and multiple linear regression analysis of circulating CD34+ cells, with relevant factors adjusted for confounding factors stratified by hypertension status. Logarithmic transformation was performed as CD34+ cells and triglycerides had a skewed distribution. All statistical analyses were performed with the SAS system for Windows (version 9.4; SAS Inc., Cary, NC). Values of p < 0.05 were regarded as statistically significant.

## Results

### Characteristics of the study population by hypertension status

Among the study population, 127 individuals were diagnosed with hypertension. Subjects with hypertension had significantly higher values of age and BMI than those without (Table 1).

**Table 1:** Characteristics of the study population by hypertension status.

	Hypertension		P
	(−)	(+)	
No. at risk	236	127	
Age (years)	65.2 ± 2.5	65.8 ± 2.9	0.044
Systolic blood pressure (mmHg)	121 ± 11	151 ± 11	<0.001
Diastolic blood pressure (mmHg)	72 ± 8	88 ± 10	<0.001
Anti-hypertensive medication use (%)	36.0	53.5	0.001
Glucose lowering medication use (%)	7.2	7.9	0.817
Lipid lowering medication use (%)	14.0	18.9	0.221
Body mass index (BMI) (kg/m^2^)	23.0 ± 2.9	23.8 ± 2.9	0.009
Current smoker (%)	24.2	21.3	0.534
Current drinker (%)	65.7	73.2	0.141
Serum triglycerides (mg/dL)	102 [71, 134]^*a^	91 [71, 127]^*a^	0.568^*b^
Serum HDL-cholesterol (HDLc) (mg/dL)	56 ± 13	57 ± 15	0.265
Hemoglobin A1c (HbA1c) (%)	5.7 ± 0.6	5.7 ± 0.6	0.773
Glomerular filtration rate (GFR) (mL/min/1.73m^2^)	71.8 ± 14.7	73.6 ± 14.5	0.275
Platelets (Plt) (×10^4^/μL)	21.9 ± 5.3	22.4 ± 4.5	0.418
CD34+ cells (cells/μL)	1.06 [0.66, 1.47]^*a^	1.04 [0.75,1.69]^*a^	0.610^*b^

Values: Mean ± standard deviation. *^a^Values are median [first quartile, third quartile]. *^b^Logarithmic transformation was used for evaluating p.

### Distribution of baseline carotid intima-media thickness (CIMT) and change in CIMT per year by hypertension status

**Figure 1:**
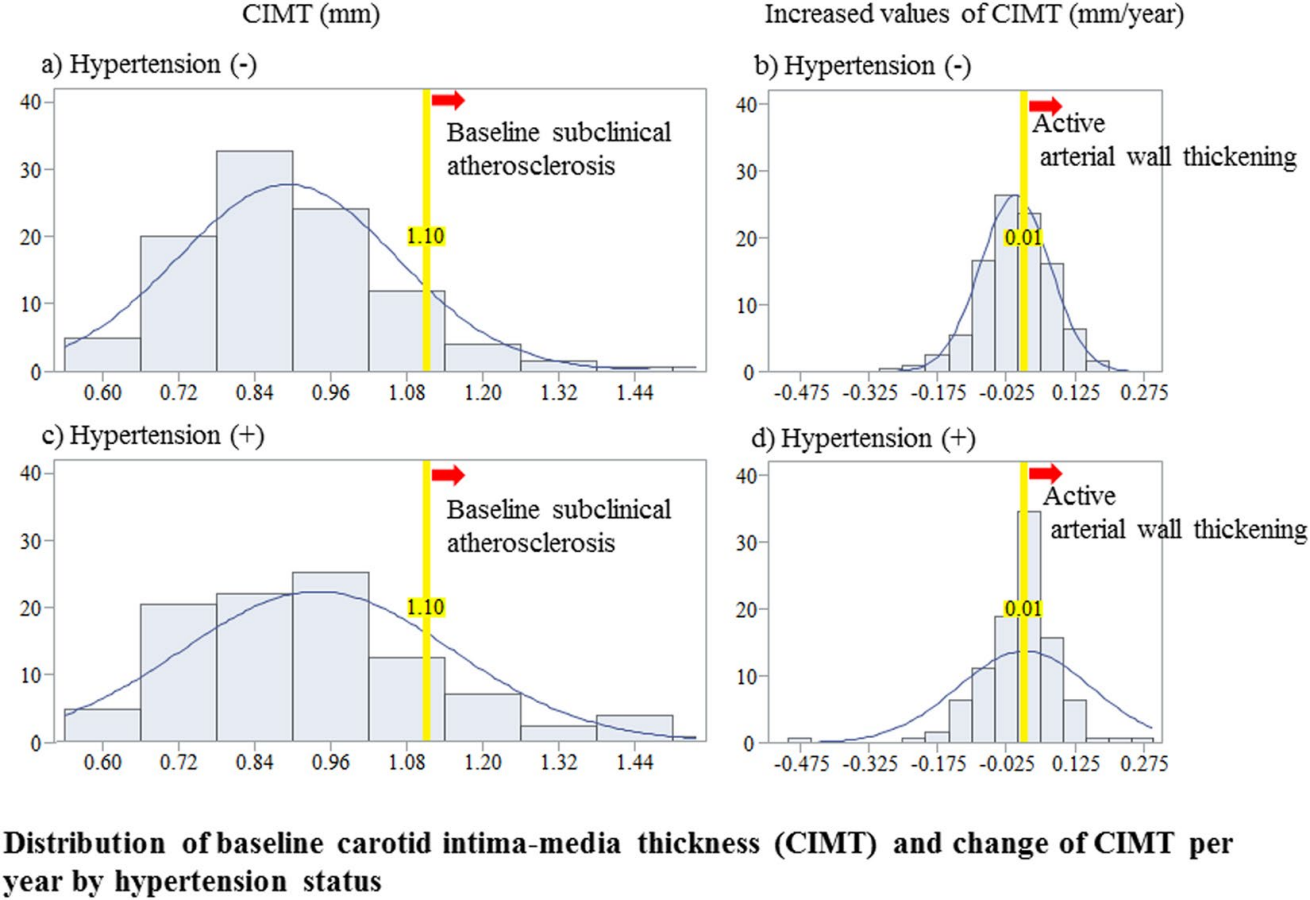
Distribution of baseline carotid intima-media thickness (CIMT) and change of CIMT per year by hypertension status.

Figure 1 shows the distribution of baseline CIMT and change in CIMT per year by hypertension status. Hypertension status did not affect those distributions.

### Baseline subclinical atherosclerosis and active arterial wall thickening in relation to hypertension

Independent of known cardiovascular risk factors, hypertension was significantly positively associated with baseline subclinical atherosclerosis. With regards to active arterial wall thickening, a positive tendency with hypertension was observed, although this was not significant (Table 2).

**Table 2:** Odds ratios (ORs) and 95% confidence intervals (CIs) for baseline subclinical atherosclerosis and active arterial wall thickening in relation to hypertension.

	Hypertension		P
	(−)	(+)	
No. at risk	236	127	
**Baseline subclinical atherosclerosis**			
No. of cases (%)	22 (9.3)	23 (18.1)	
Model 1	1.00	2.15 (1.14, 4.05)	0.018
Model 2	1.00	2.16 (1.43, 4.07)	0.018
Model 3	1.00	2.41 (1.28, 4.78)	0.007
**Active arterial wall thickening**			
No. of cases (%)	102 (43.2)	62 (48.8)	
Model 1	1.00	1.21 (0.78, 1.87)	0.389
Model 2	1.00	1.24 (0.79, 1.90	0.367
Model 3	1.00	1.22 (0.78, 1.92)	0.376

Model 1: Adjusted only for age. Model 2: Further adjusted for smoking status (never-smoker, former smoker, or current smoker). Model 3: Model 2 + further adjusted for body mass index, and alcohol consumption (never-drinker, former drinker, or current drinker [23–45 g/week, 46–68 g/week, ≥69 g/week, respectively]), HDL-cholesterol, triglycerides, HbA1C, and GFR. Active arterial wall thickening was defined as an increment of carotid intima-media thickness (CIMT) ≥ 0.01 mm/year. Baseline subclinical atherosclerosis was defined as CIMT ≥ 1.1 mm.

### Active arterial wall thickening in relation to baseline subclinical atherosclerosis

Independent of known cardiovascular risk factors, baseline subclinical atherosclerosis is significantly inversely associated with active arterial wall thickening (Table 3).

**Table 3:** Odds ratios (ORs) and 95% confidence intervals (CIs) for active arterial wall thickening in relation to baseline subclinical atherosclerosis.

	Baseline subclinical atherosclerosis		P
	(−)	(+)	
No. at risk	318	45	
No. of cases (%)	155 (48.7)	9 (20.0)	
Model 1	1.00	0.26 (0.12, 0.56)	<0.001
Model 2	1.00	0.26 (0.12, 0.57)	<0.001
Model 3	1.00	0.24 (0.11, 0.52)	<0.001

Model 1: Adjusted only for age. Model 2: Further adjusted for smoking status (never-smoker, former smoker, or current smoker). Model 3: Model 2 + further adjusted for body mass index, systolic blood pressure, and alcohol consumption (never-drinker, former drinker, or current drinker [23–45 g/week, 46–68 g/week, ≥ 69 g/week, respectively]), HDL-cholesterol, triglycerides, HbA1C, and GFR. Active arterial wall thickening was defined as an increment of carotid intima-media thickness (CIMT) ≥ 0.01 mm/year.

Baseline subclinical atherosclerosis was defined as CIMT ≥ 1.1 mm.

### CD34+ cell count by endothelium status

Table 4 shows the CD34+ cell count by endothelium status. There was no significant difference between those with and without baseline subclinical atherosclerosis. However, subjects with active arterial wall thickening had significantly higher CD34+ cell counts than those without active arterial wall thickening.

**Table 4:** CD34+ cell count by endothelium status.

	Baseline subclinical atherosclerosis		p	Active arterial wall thickening		p
	(−)	(+)		(−)	(+)	
No. of cases	318	45		199	164	
CD34+ cells (cells/µL)	1.05 [0.67, 1.56]^*a^	1.06 [0.79, 1.69]*^a^	0.130*^b^	0.95 [0.63, 1.53]*^a^	1.12 [0.76, 1.61]*^a^	0.047*^b^

^*a^Values are median [first quartile, third quartile]. *^b^Logarithmic transformation was used for evaluating p.

### Active arterial wall thickening in relation to circulating CD34+ cells by hypertension status

In Model 1, among non-hypertensive subjects, there was no clear linear relationship between circulating CD34+ cells and active arterial wall thickening. After further adjustments for smoking status (Model 2), this relationship became clearly linear; this linear association remained unchanged after further adjustments for other known cardiovascular risk factors (Model 3). No significant relationships were observed among hypertensive subjects (Table 5).

**Table 5:** Odds ratios (ORs) and 95% confidence intervals (CIs) for active arterial wall thickening in relation to circulating CD34+ cell count.

	Circulating CD34+ cell				P	CD34+ cell (logarithmic values)
	Low (Q1)	Q2	Q3	High (Q4)		
**Non- hypertension**						
No. at risk	64	53	65	54		
No. of cases (%)	17 (26.6)	26 (49.1)	31 (47.7)	28 (51.9)		
Model 1	1.00	2.63 (1.21, 5.73)	2.59 (1.23, 5.45)	3.00 (1.38, 6.50)	0.007	1.85 (1.23, 2.78)
Model 2	1.00	2.75 (1.25, 6.04)	3.19 (1.47, 6.92)	3.34 (1.51, 7.37)	0.003	1.93 (1.27, 2.93)
Model 3	1.00	2.69 (1.22, 5.95)	2.98 (1.35, 6.56)	3.01 (1.31, 6.94)	0.009	1.83 (1.19, 2.84)
**Hypertension**						
No. at risk	27	37	25	38		
No. of cases (%)	14 (51.9)	18 (48.6)	14 (56.0)	16 (42.1)		
Model 1	1.00	0.87 (0.32, 2.34)	1.17 (0.39, 3.50)	0.67 (0.25, 1.80)	0.503	0.70 (0.38, 1.29)
Model 2	1.00	0.88 (0.32, 2.39)	1.17 (0.39, 3.51)	0.67 (0.25, 1.80)	0.497	0.70 (0.38, 1.29)
Model 3	1.00	0.79 (0.28, 2.21)	1.13 (0.36, 3.53)	0.63 (0.22, 1.81)	0.512	0.69 (0.36, 1.32)

Model 1: Adjusted only for age. Model 2: Further adjusted for smoking status (never-smoker, former smoker, or current smoker). Model 3: Model 2 + further adjusted for body mass index, systolic blood pressure, and alcohol consumption (never-drinker, former drinker, or current drinker [23–45 g/week, 46–68 g/week, ≥69 g/week, respectively]), HDL-cholesterol, triglycerides, HbA1C, and GFR. Active arterial wall thickening was defined as an increment of carotid intima-media thickness (CIMT) ≥ 0.01 mm/year.

### Effect of the modification of hypertension on the association between active arterial wall thickening and circulating CD34+ cells

As the significant positive association between active arterial wall thickening and circulating CD34+ cells was only observed in subjects without hypertension, we tested the effect of differing hypertension status on the association between active arterial wall thickening and circulating CD34+ cells, and found significant differences using all models: p < 0.001 for Model 1, p = 0.009 for Model 2, and p = 0.009 for Model 3 (data not shown).

### Association between circulating CD34+ cells and platelets by hypertension

As the combination of platelets and circulating CD34+ cells could act as an indicators of aggressive endothelial repair, which relates to wasting reduction of CD34+ cells^6,34^, we also evaluated the association between circulating CD34+ cells and platelets. The simple correlation coefficient showed a significant positive association between circulating CD34+ cells and platelets only for subjects without hypertension. These associations remained unchanged even after adjustments for other possible confounding factors such as age, systolic blood pressure, current smoker, current drinker [≥23 g/week], BMI (kg/m^2^), HDLc (mg/dL), triglycerides (mg/dL), HbA1c (%), and GFR (mL/min/1.73 m^2^) (Table 6).

**Table 6:** Simple correlation analysis and multiple linear regression analysis of circulating CD34+ cells with relevant factors adjusted for confounding factors by hypertension status.

	Hypertension							
	(−)				(+)			
	r (p)	Β	β	P	r (p)	Β	β	p
No. at risk	236				127			
Age	−0.001 (0.993)	0.01	0.02	0.716	0.001 (0.992)	0.01	0.02	0.807
Systolic blood pressure	0.08 (0.215)	0.0001	0.001	0.990	−0.01 (0.912)	0.001	0.02	0.865
Current smoker	0.05 (0.426)	−0.07	−0.02	0.719	−0.08 (0.349)	−0.18	−0.10	0.296
Current drinker	0.02 (0.807)	−0.18	−0.07	0.339	−0.11 (0.222)	−0.21	−0.12	0.188
BMI	0.24 (<0.001)	0.07	0.16	0.024	0.16 (0.723)	0.02	0.09	0.341
HDL-cholesterol	−0.18 (0.005)	0.003	0.03	0.670	−0.18 (0.050)	−0.01	−0.10	0.319
Triglycerides	0.26 (<0.001)	0.55	0.21	0.003	0.16 (0.078)	0.14	0.10	0.326
HbA1C	0.14 (0.030)	0.04	0.02	0.780	0.07 (0.455)	0.07	0.05	0.563
GFR	0.02 (0.816)	0.001	0.01	0.875	0.13 (0.152)	0.01	0.13	0.162
Platelet	0.28 (<0.001)	0.05	0.23	<0.001	0.17 (0.054)	0.02	0.15	0.105

r (p): Simple correlation coefficient (p value). Β: Parameter estimate. β: Standardized parameter estimate. p: p value for multivariable linear regression models. BMI: Body mass index. GFR: Glomerular filtration rate. Triglycerides and circulating CD34+ cells were calculated as logarithmic values.

For sensitivity analysis, we reran the main results by defining active arterial wall thickening as mean values of CIMT ≥ 1.1 mm and found essentially the same associations, although this association did not reach significance. The fully adjusted ORs of active arterial wall thickening evaluated based on the mean value of CIMT (n = 91 for non-hypertension and n = 55 for hypertension) for the logarithmic circulating CD34+ cell count were 1.26 (0.83, 1.91) and 0.81 (0.42, 1.59) for subjects without and with hypertension, respectively.

## Discussion

The major findings of the present study were that, in elderly men without hypertension, the CD34+ cell count was significantly positively associated with active arterial wall thickening, but not for subjects with hypertension. These results provide a means to clarify the underlying mechanisms of endothelium repair in elderly subjects.

Bone marrow-derived hematopoietic progenitor cells (immature cells such as CD34+ cells) have been revealed to play a major role in vascular maintenance^1,2,25,36^. Hematopoietic progenitor cells have been observed in human atherosclerotic lesions^37,38^, and increased hematopoietic activity has also been reported in patients with atherosclerosis^39^. Furthermore, CD34+ cells are reported to differentiate not only into endothelial cells, but also foam cells^25^. Therefore, a higher number of circulating CD34+ cells indicates that there is enough material for atherosclerosis to develop (active arterial wall thickening). We found a significant positive association between active arterial wall thickening and circulating CD34+ cells. The fact that our previous cross-sectional study showed a positive association between CIMT and chronic kidney disease (CKD), limited to subjects with a high number of circulating CD34+ cells, also supports this mechanism^40^ since glomerular injury and atherosclerosis (increased arterial stiffness) has a common initial process of endothelial dysfunction^41^. This previous study also supports the theory that higher numbers of circulating CD34+ cells indicates that there is enough material for atherosclerosis to develop (active arterial wall thickening).

Our other previous cross-sectional studies with hypertensive subjects indicates that subjects who do not experience progression of sarcopenia may possess the capacity for active endothelial repair that causes atherosclerosis, as evaluated by CIMT^26,27^. These studies indicate that active arterial wall thickening should have a beneficial influence on vascular maintenance because the age-related disruption of the micro endothelium and the impairment of blood flow exacerbates both hypertension and sarcopenia^42^. However, in the present study, the positive association between circulating CD34+ cells and active arterial wall thickening is observed only in subjects without hypertension.

A wasting reduction in circulating CD34+ cells may act as a strong confounding factor in the association between circulating CD34+ cells and active arterial wall thickening for subjects with hypertension.

Because of aggressive endothelial repair that causes atherosclerosis progression, several CD34+ cells were differentiated into mature cells, such as endothelial, mural, and foam cells^2,25^. Since these mature cells are known as CD34- (negative) cells, wasting reduction in circulating CD34+ cells might be caused by aggressive endothelial repair related to atherosclerosis^13^.

Hypertension induces aggressive endothelial repair by injuring the endothelium. Therefore, subjects with hypertension should have stronger wasting reduction in circulating CD34+ cells than those without hypertension. In our study, hypertension was revealed to be significantly positively associated with baseline subclinical atherosclerosis, indicating aggressive endothelial repair. In addition, platelet count was significantly positively correlated with the number of circulating CD34+ cells (r = 0.28, p ≤ 0.001) in subjects without hypertension, whereas no significant correlation was observed in subjects with hypertension (r = 0.17, p = 0.054).

Platelets contribute to endothelial repair (vascular maintenance) in conjunction with CD34+ cells^17,25,34^. However, the ratio of the decrease in the platelet count due to their use in endothelial repair might be much smaller than that of circulating CD34+ cells in patients with hypertension. Therefore, our previous cross-sectional study showed that platelet count was significantly positively associated with circulating CD34+ cells in subjects without hypertension, whereas no significant association was observed for subjects with hypertension. In addition, this previous study revealed that platelet count was not significantly associated with CIMT among subjects without hypertension but that it significantly positively associated with CIMT among those with hypertension^34^.

These findings support the above-mentioned mechanism, showing that the status of hypertension could be associated with wasting reduction of circulating CD34-positive cells.

Furthermore, subjects with baseline subclinical atherosclerosis should induce wasting reduction of circulating CD34+ cell which leads to a deficit of the source material for atherosclerosis development (active arterial wall thickening). Therefore, the existence of subclinical atherosclerosis at baseline significantly lowered the risk of active arterial wall thickening in the present study. We also found that hypertension showed no significant association with active arterial wall thickening.

These results also confirmed that subjects with hypertension should have a higher risk of endothelial injury, promoting the positive association between hypertension and subclinical atherosclerosis. Subjects with hypertension had higher risks of wasting reduction of circulating CD34+ cells^6^ that disrupt the positive association between hypertension and active arterial wall thickening.

**Figure 2:**
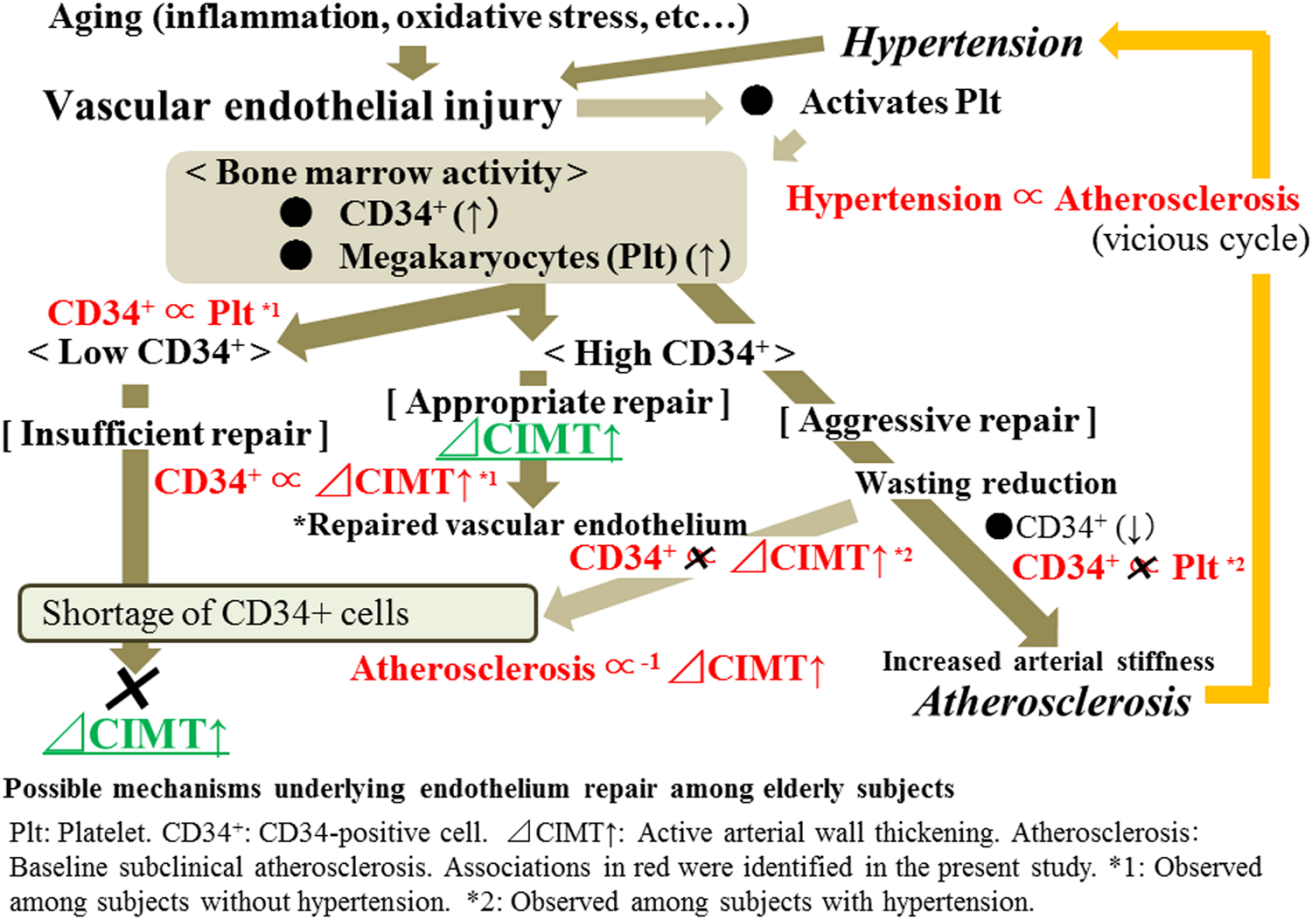
Possible mechanisms underlying endothelium repair among elderly subjects.

The summary of the possible underlying mechanisms of endothelium repair among elderly subjects is shown in Fig. 2. Briefly, the levels of subendothelial components that stimulate platelet activation are elevated in peripheral blood following injury to the endothelium^43,44^. Activated platelets elevate circulating CD34-positive cells by promoting mobilization from the bone marrow and acting as a bridge between damaged vessels and CD34+ cells^17–21,45^. The number of platelets is much higher than the number of CD34+ cells. Therefore, both the production and wasting reduction determine the numbers of circulating CD34+ cell, while mainly production, with only a limited influence of wasting reduction, determines the numbers of platelets. Moreover, hypertension, which is positively associated with baseline subclinical atherosclerosis, could also be associated with the wasting reduction of circulating CD34+ cells.

The clinical implication of the present study is that the absence of active arterial wall thickening is rarely indicative of a low-risk of endothelial dysfunction among elderly subjects. Instead, a wasting reduction of hematopoietic progenitor cells may be the reason for this phenomenon.

There are several strengths of the present study. First, our study is the largest general population-based prospective study in the world that deals with circulating CD34+ cells in a strict manner. Second, the target population was limited to a narrow age range (60–69 years), and the study only included men since the vascular maintenance capacity is considered to be strongly influenced by gender difference and age. Third, we used the Intimascope to measure CIMT, which has a 10-fold higher axial resolution, at an estimated scale of 0.01 mm^32^, which enables us to evaluate active arterial wall thickening with a high accuracy. Furthermore, dissimilar to typical epidemiological studies, our study used multi-faceted analyses that could support the possible mechanisms underlying the major findings.

There are also a number of potential limitations of the present study. First, although we believe that active arterial wall thickening provides a beneficial influence on vascular endothelial maintenance in elderly subjects, as we observed in our previous cross-sectional study^26,27^, we have no data in which to evaluate this beneficial influence. As such, further prospective study, including the data of CIMT and muscle strength, is necessary. Second, we have no data regarding the factors that injure the endothelium directly, even those which stimulate endothelial repair. In order to enhance the findings of the current research, further prospective study with oxidative stress is also necessary. Moreover, we have no knowledge of the exact cut-off point for evaluating active arterial wall thickening when using the Intimascope, which might cause misclassification. However, the present study used a multi-faceted analysis that explains the potential background of the major results. Furthermore, sensitivity analysis, which defined active arterial wall thickening based on mean CIMT values, shows essentially the same associations.

In conclusion, for elderly subjects without hypertension, the CD34+ cell count is significantly positively associated with active arterial wall thickening, but not for elderly subject with hypertension. These results demonstrate an efficient tool to clarify the underlying mechanisms of endothelium repair in elderly subjects.

## References

[1] Shi Q (1998). Evidence for circulating bone marrow-derived endothelial cells. Blood.

[2] Yamada Y, Takakura N (2006). Physiological pathway of differentiation of hematopoietic stem cell population into mural cells. J. Exp. Med..

[3] Asahara T (1997). Isolation of putative progenitor endothelial cells for angiogenesis. Science..

[4] Bielak LF (2009). Circulating CD34+ cell count is associated with extent of subclinical atherosclerosis in asymptomatic Amish men, independent of 10-year Framingham risk. Clin. Med. Cardiol..

[5] Shimizu Y (2015). Circulating CD34-positive cells, glomerular filtration rate and triglycerides in relation to hypertension. Atherosclerosis.

[6] Shimizu Y (2017). Platelets and circulating CD34-positive cells as an indicator of the activity of the vicious cycle between hypertension and endothelial dysfunction in elderly Japanese men. Atherosclerosis.

[7] Brusnahan SK (2010). Human blood and marrow side population stem cell and Stro-1 positive bone marrow stromal cell numbers decline with age, with an increase in quality of surviving stem cells: correlation with cytokines. Mech. Ageing Dev..

[8] Garvin K, Feschuk C, Sharp JG, Berger A (2007). Does the number or quality of pluripotent bone marrow stem cells decrease with age?. Clin. Orthop. Relat. Res..

[9] Guralnik, J. M., Ershler, W. B., Schrier, S. L. & Picozzi, V. J. Anemia in the elderly: a public health crisis in hematology. *Hematology Am Soc Hematol Educ Program*, 528–532 (2005). 10.1182/asheducation-2005.1.528 16304431

[10] Cooper B (2011). The origins of bone marrow as the seedbed of our blood: from antiquity to the time of Osler. Proc (Bayl Univ Med Cent)..

[11] Ungvari Z, Tarantini S, Donato AJ, Galvan V, Csiszar A (2018). Mechanisms of vascular aging. Cir. Res..

[12] Rea IM (2018). Age and age-related disease: role of inflammation triggers and cytokines. Front. Immunol..

[13] Shimizu Y (2018). Hepatocyte growth factor and carotid intima-media thickness in relation to circulating CD34-positive cell levels. Environ Health. Prev Med..

[14] Shimizu Y (2019). Gamma-glutamyl transpeptidase (γ-GTP) has an ambivalent association with hypertension and atherosclerosis among elderly Japanese men: a cross-sectional study. Environ Health. Prev Med..

[15] Shi G, Morrell CN (2011). Platelets as initiators and mediators of inflammation at the vessel wall. Thromb. Res..

[16] Ombrello C, Block RC, Morrell CN (2010). Our expanding view of platelet functions and its clinical implications. J. Cardiovasc. Transl. Res..

[17] Stellos K (2008). Platelet-derived stromal cell-derived factor-1 regulates adhesion and promotes differentiation of human CD34+ cells to endothelial progenitor cells. Circulation.

[18] Stellos K (2009). Expression of stromal-cell-derived factor-1 on circulating platelets is increased in patients with acute coronary syndrome and correlates with the number of CD34+ progenitor cells. Eur. Heart J..

[19] Seitz G, Boehmler AM, Kanz L, Möhle R (2005). The role of sphingosine 1-phosphate receptors in the trafficking of hematopoietic progenitor cells. Ann. N. Y. Acad. Sci..

[20] Golan K (2012). S1P promotes murine progenitor cell egress and mobilization via S1P1-mediated ROS signaling and SDF-1 release. Blood.

[21] Zou J, Yuan C, Wu C, Cao C, Yang H (2014). The effects of platelet-rich plasma on the osteogenic induction of bone marrow mesenchymal stem cells. Connect. Tissue Res..

[22] Matsubara Y (2013). OP9 bone marrow stroma cells differentiate into megakaryocytes and platelets. PLoS One.

[23] Gunn N (2003). High CD34+ cell dose promotes faster platelet recovery after autologous stem cell transplantation for acute myeloid leukemia. Biol. Blood Marrow Transplant..

[24] Lindemann S, Krämer B, Seizer P, Gawaz M (2007). Platelets, inflammation and atherosclerosis. J. Thromb. Haemost..

[25] Daub K (2006). Platelets induce differentiation of human CD34+ progenitor cells into foam cells and endothelial cells. FASEB J..

[26] Shimizu Y (2017). Handgrip strength and subclinical carotid atherosclerosis in relation to platelet levels among hypertensive elderly Japanese. Oncotarget.

[27] Shimizu Y (2018). Association between tongue pressure and subclinical carotid atherosclerosis in relation to platelet levels in hypertensive elderly men: a cross-sectional study. Environ Health. Prev Med..

[28] National Institute of Population and Social Security Research [Home page on the Internet]. http://www.ipss.go.jp/pp-shicyoson/j/shicyoson13/3kekka/Municipalities.asp [Cited March 2. 2020].

[29] Sutherland DR, Anderson L, Keeney M, Nayar R, Chin-Yee I (1996). The ISHAGE guidelines for CD34+ cell determination by flow cytometry. International Society of Hematotherapy and Graft Engineering. J. Hematother.

[30] Imai E (2009). Prevalence of chronic kidney disease in the Japanese general population. Clin. Exp. Nephrol..

[31] Hara T (2006). Evaluation of clinical markers of atherosclerosis in young and elderly Japanese adults. Clin. Chem. Lab. Med..

[32] Yanase T (2006). Evaluation of a new carotid intima-media thickness measurement by B-mode ultrasonography using an innovative measurement software, intimascope. Am. J. Hypertens..

[33] Kawamori R (1992). Prevalence of carotid atherosclerosis in diabetic patients. Ultrasound high-resolution B-mode imaging on carotid arteries. Diabetes Care.

[34] Shimizu Y (2016). Platelets as an indicator of vascular repair in elderly Japanese men. Oncotarget.

[35] Shimizu Y (2019). Consumptive reduction following increased production of CD34-positive cells and carotid intima-media thickness in non-hypertensive elderly Japanese men. Cogent Medicine..

[36] Takakura N (2000). A role for hematopoietic stem cells in promoting angiogenesis. Cell.

[37] Torsney E, Mandal K, Halliday A, Jahangiri M, Xu Q (2007). Characterisation of progenitor cells in human atherosclerotic vessels. Atherosclerosis.

[38] Moreno PR (2004). Plaque neovascularization is increased in ruptured atherosclerotic lesions of human aorta: implications for plaque vulnerability. Circulation.

[39] van der Valk FM (2017). Increased haematopoietic activity in patients with atherosclerosis. Eur. Heart J..

[40] Shimizu Y (2019). Association between chronic kidney disease and carotid intima-media thickness in relation to circulating CD34-positive cell count among community-dwelling elderly Japanese men. Atherosclerosis.

[41] Endemann DH, Schiffrin EL (2004). Endothelial dysfunction. J. Am. Soc. Nephrol..

[42] Payne GW (2006). Effect of inflammation on the aging microcirculation: impact on skeletal muscle blood flow control. Microcirculation.

[43] Wu KK, Phillips M, D’Souza D, Hellums JD (1994). Platelet activation and arterial thrombosis. Lancet.

[44] Nakamura T, Kambayashi J, Okuma M, Tandon NN (1999). Activation of the GP IIb -IIIa complex induced by platelet adhesion to collagen is mediated by both alpha2beta1 integrin and GP VI. J. Biol. Chem..

[45] Cangiano E (2011). Different clinical models of CD34+ cells mobilization in patients with cardiovascular disease. J. Thromb. Thrombolysis.

